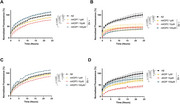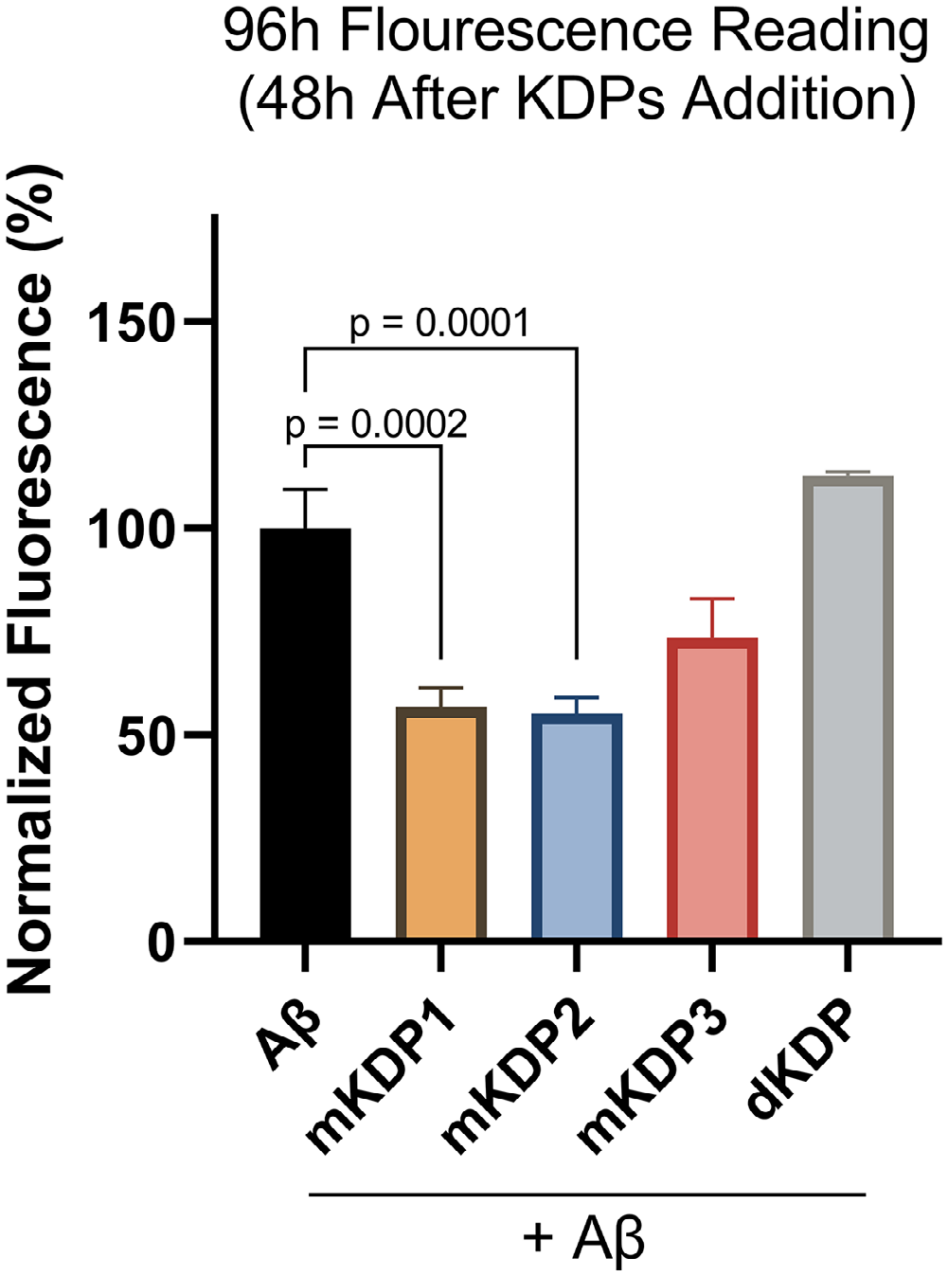# Anti Amyloid‐Beta Aggregation Activity of Kefir‐Derived Peptides

**DOI:** 10.1002/alz70859_103539

**Published:** 2025-12-25

**Authors:** Lucas Matos Martins Bernardes, Serena Mares Malta, Matheus Henrique Silva, Ana Carolina Costa Santos, Tamiris Sabrina Rodrigues, Fernanda Araújo do Prado Mascarenhas, Renata Graciele Zanon, Foued Salmen Espindola, Ana Paula Mendes‐Silva, Carlos Ueira‐Vieira

**Affiliations:** ^1^ Universidade Federal de Uberlândia, Uberlândia, Minas Gerais Brazil; ^2^ University of Saskatchewan, Saskatoon, SK Canada

## Abstract

**Background:**

Kefir is a fermented beverage rich in beneficial probiotics, and its water‐soluble <10kDa fraction has demonstrated antioxidant activity, acetylcholinesterase inhibition, and neuroprotection in *Drosophila melanogaster* Alzheimer’s disease (AD) models. Using in silico mutagenesis, we designed mutated versions of two kefir‐derived peptides (KDPs) to enhance their binding affinity to amyloid‐beta (Aβ) and their potential to cross the blood‐brain barrier (BBB). We then evaluated their effectiveness in preventing or disrupting Aβ plaque formation in vitro.

**Method:**

KDPs were mutated using ToxinPred. ExPASy PeptideCutter yielded digested KDPs (dKDPs). Bioactivity and BBB permeability were predicted with PeptideRanker and BBPpred. Mutated KDPs (mKDPs) and dKDPs were docked with Aβ monomers using ClusPro. Top mKDPs (1, 2, 3) and dKDP were synthesized and tested in a thioflavin T aggregation assay. For early treatment, peptides (1, 10 and 100 µM) were added with Aβ, and fluorescence was measured hourly for 24h. For late treatment, peptides (10 µM) were added after 48h of the addition of Aβ, with a reading at 96h. Statistical analysis used repeated measures one‐way ANOVA.

**Result:**

In early treatment, mKDP1 reduced Aβ aggregation by 23%, mKDP2 by 56%, mKDP3 by 16%, and dKDP by 57% after 24 hours (p<0.0001 for all comparisons). In late treatment, mKDP1 and mKDP2 reduced Aβ aggregation by approximately 45% (p=0.0002 and p=0.0001, respectively), while mKDP3 and dKDP showed no significant effects.

**Conclusion:**

All peptides showed anti‐Aβ aggregation effects in early administration, and mKDP1 and mKDP2 in late stages. Further in vivo studies are needed to validate these findings.